# Nitrogen-doped hollow carbon sphere composite Mn_3_O_4_ as an advanced host for lithium-sulfur battery

**DOI:** 10.1038/s41598-024-64067-8

**Published:** 2024-06-14

**Authors:** Haibin Wang, Jun Liu, Wenqi Ju, Xupeng Xu, Jiwei Chen

**Affiliations:** 1 School of Materials Science and Engineering, Hunan Institute of Technology, Hengyang, 421002 China; 2 School of Materials Science and Engineering, Xiangtan University, Hunan, 411105 China

**Keywords:** Lithium-sulfur batteries, Mn_3_O_4_ particles, Hollow carbon microsphere, Energy science and technology, Materials science

## Abstract

As the most promising advanced energy storage system, lithium-sulfur batteries (LSBs) are highly favored by the researchers because of their advantages of high energy density (2500 W h kg^−1^), low cost and non-pollution. However, the low conductivity, volume expansion of sulfur, and shuttle effect are still the great hindrance to the practical application of LSBs. Herein, the above problems can be addressed through the following strategies: (1) Hollow carbon microspheres with high specific surface area were constructed as sulfur hosts to increase sulfur loading while also being able to enhance the physical adsorption of polysulfides; (2) the loading of Mn_3_O_4_ particles on the basis of hollow carbon microspheres facilitates the capture and adsorption of polysulfides; (3) the hollow carbon sphere structure as a conductive network can provide more pathways for rapid electrical/ionic transport and also accelerate electrolyte wetting. Moreover, the thinner shell of hollow carbon microsphere is conducive to ion diffusion and speed up the reaction rate. Thus, the NHCS/Mn_3_O_4_/S composites exhibit a high discharge specific capacity of 1010.3 mAh g^−1^ at first and still maintained a reversible capacity of 269.2 mAh g^−1^ after 500 cycles. This work presents a facile sustainable and efficient synergistic strategy for the development of advanced LSBs.

## Introduction

With the rapid development of energy storage systems, new high energy density battery systems have been widely studied^1^. LSBs have extremely high theoretical specific capacity (1675 mAh g^−1^) and energy density (2500 Wh kg^−1^), making them ideal for a new generation of energy storage systems^2^. However, the low conductivity, volume expansion of sulfur of sulfur, and shuttle effect of polysulfides are still major challenges before the commercial application of LSBs^3,4^.

Using carbon material as a sulfur host can effectively alleviate the above problems. Among them, the effect is particularly obvious when using porous^5^, carbon nanotubes^6^, carbon fibers^7^ and porous biochar^8^ with high porosity and good electrical conductivity. These materials can greatly improve the electron exchange ability of sulfur in electrochemical reactions, and can also play a good physical adsorption effect on polysulfide. When metal compounds such as titanium dioxide^9^, manganese oxide^10^ and aluminum fluoride^11^ are used as hosts, they can also play a certain chemisorption effect on polysulfides, thus alleviating the shuttle effect to a certain extent. However, neither physical adsorption nor chemical adsorption can effectively alleviate the shuttle of polysulfide. Therefore, researchers usually design reasonable structures that combine the dual effects of physical adsorption and chemisorption as base materials for sulfur^12^. The prepared cathode material can improve the overall conductivity of the positive electrode and can capture and store soluble polysulfide, which can effectively alleviate the shuttle effect^13,14^.

Based on this, we used silica nanospheres with a diameter of about 300 nm as the hard template, dopamine hydrochloride as the carbon source and nitrogen source, and successfully prepared nitrogenous hollow carbon spheres (NHCS) by carbonizing at 800℃ and removing the template by HF. Then, the NHCS was combined with Mn_3_O_4_ as the sulfur host by hydrothermal method. The material has the following advantages: (1) the NHCS is hollow spherical, the size distribution is uniform, the diameter is about 300 nm, has a high specific surface area (422 m^2^ g^−1^), has the potential to hold more active sulfur, and enhances the physical adsorption of polysulfide; (2) Mn_3_O_4_ and N elements have strong chemisorption and provide more active sites, which is conducive to the adsorption and conversion of lithium ions. (3) The construction of a hollow carbon sphere structure forms a conductive network with good conductivity, which provides more ways for the rapid transmission of electricity/ions and can accelerate the penetration of electrolytes. The thinner shell is conducive to ion diffusion and speed up the reaction rate. The synergistic effect of the hollow carbon shell frame and N and Mn_3_O_4_ can alleviate the volume expansion effect, increase the sulfur load, significantly improve the conductivity of carbon materials, and effectively alleviate the shuttle effect of LSBs.

## Experimental

### Preparation of nitrogen-containing hollow carbon spheres (NHCS)

First, as a hard template, 2.0 g of silica gel spheres with a diameter of about 300 nm were weighed. And then, 180 mL deionized water, 80 mL ethanol and SiO_2_ powder were mixed and stirred for 1 h. Then, the mixture was transferred to the ultrasonic dispersion instrument for ultrasonic dispersion for 1 h. 6 mL ammonia water was then added into the above mixture, stirred for 30 min at 30 ℃. Take 20 mL PDA solution with a concentration of 50 g L^−1^ and add it to the mixture prepared previously. After continuous stirring for 24 h at room temperature, the compound substance was washed and filtered for several times to obtain precursor powder of polydopamine-coated silica composite. The composite was then dried in an oven for 12 h at 60 ℃ to obtain PDA coated SiO_2_ powder (SiO_2_@PDA). SiO_2_@PDA was placed in a tubular furnace and heated to 800 ℃ for 3 h with a heating rate of 5 °C min^−1^ under nitrogen atmosphere and the SiO_2_@NHCS obtain. Finally, NHCS was obtained by etching with 10% HF solution for 12 h.

### Preparation of composite materials NHCS/S and NHCS/Mn_3_O_4_/S

**Figure 1:**
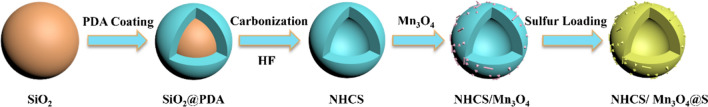
Preparation process of NHCS/Mn_3_O_4_@S composites.

The whole preparation model is shown in Fig. 1. Firstly, the prepared NHCS (0.2 g) was added to 50 mL deionized water and mixed in a beaker, ultrasonically dispersed for 2 h, and then 0.078 g KMnO_4_ and 0.123 g urea were added to the mixture successively. Secondly, after 20 min of intense stirring, the mixture was transferred to a high pressure reactor for a hydrothermal reaction for 6 h at 120 °C. The powder obtained by hydrothermal reaction was washed with deionized water and alcohol in sequence, and then dried for 12 h at 150 ℃ to obtain NHCS/Mn_3_O_4_. Finally, NHCS/Mn_3_O_4_@S and NHCS@S nanocomposites were obtained after NHCS and NHCS/Mn_3_O_4_ and sublimed sulfur were fully mixed in agate mortar for 20 min at the mass ratio of 3:7, and then transferred to a vacuum reactor, heated for 12 h at 155 °C, and cooled down to room temperature.

### Characterizations

The crystalline structures of NPCS/Mn_3_O_4_, NPCS/Mn_3_O_4_/S and S ware characterized by XRD using a diffractometer (Rigaku, Cu Ka radiation) from 5° to 85°. The surface areas and pore sizes of NPCS and NPCS/Mn_3_O_4_ were estimated by the Brunauer–Emmett–Teller (BET) and Barrett-Joyner-Halenda (BJH) methods, respectively. Pyris 1 TGA (PerkinElmer, USA) was used to test thermogravimetric curves with a heating rate of 10 °C min^−1^ from room temperature to 800 °C in a nitrogen atmosphere. The morphology, microstructure, and element components of different samples were characterized by scanning electron microscope (SEM) and energy-dispersive X-ray spectroscopy (EDS). High-resolution transmission electron microscopy (TEM) (TecnaiG2 F20 S-TWIN microscope) was used to obtain the structure of the prepared materials. X-ray photoelectron spectroscopy (XPS) patterns were obtained on a Scientific K-Alpha instrument to investigate the chemical state of samples.

### Electrochemical responses

The electrodes were prepared by mixing S contained active material, conductive material of Super-P, and polyvinylidene fluoride (PVDF) with a ratio of 7:2:1. All electrochemical properties of the electrodes were tested by assembling CR2025 coin-type half-cells. The actual sulfur content of the electrodes is about 1 mg cm^−2^ and the actual amount of electrolyte in each cell is about 30 μL.The electrolyte was lithium bis(trifluorome-thanesulfonyl) imid (1 M L^−1^) dissolved in 1, 3-dioxolane (DOL) and 1, 2-dimethoxyethane (DME) in a volume ratio of 1:1 with lithium nitrate (LiNO_3_, 0.2 M L^−1^) as the main salt. Constant current charge/discharge tests were carried out over a voltage range of 1.7–2.8 V using the BTS-XWJ-6.44S-00052 model Neware Battery Test System. Cyclic voltammetry and AC impedance tests were performed on electrochemical workstation (CHI660a). The cyclic voltammetric voltage was in the range of 1.7–2.8 V at a scanning rate of 0.1 mV s^−1^. The AC impedance frequency range was 10^–2^–10^5^ Hz and the amplitude was ± 5 mV. The sulfur mass in the electrode was measured and estimated to determine the current density and specific capacity in the experiment.

## Results and discussion

### Morphology and structure of materials

**Figure 2:**
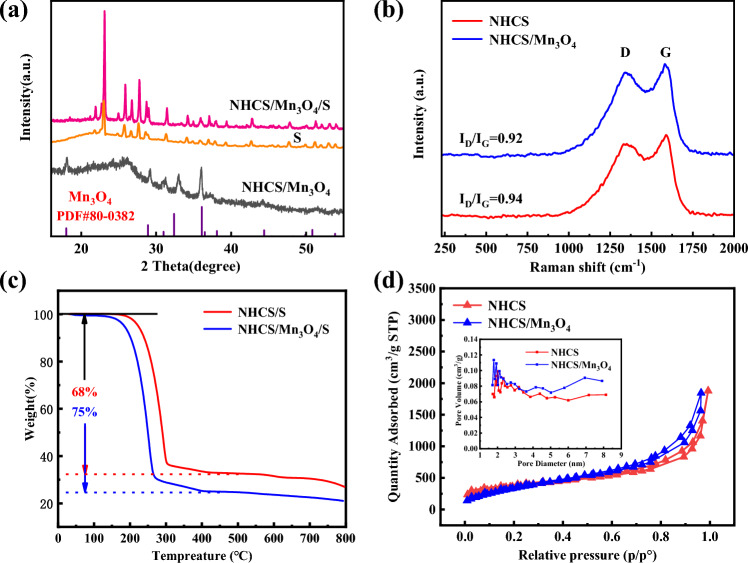
XRD patterns of (**a**) S, NHCS/Mn_3_O_4_, NHCS/Mn_3_O_4_@S; (**b**) Raman diagrams of NHCS and NHCS/Mn_3_O_4_; (**c**) TGA curves of NHCS and NHCS/Mn_3_O_4_; (**d**) N_2_ adsorption/desorption isotherm and pore size distribution diagram (illustration).

Figure 2a is the XRD diffraction pattern of each sample. It can be seen that the NPCS/Mn_3_O_4_ sample has a relatively obvious characteristic peak at 26° corresponding to the (002) crystal face of graphitic carbon, indicating that the nitrogen-containing hollow carbon sphere has a good degree of graphitization and good electrical conductivity. The spectral lines also correspond well with the card characteristic peaks of Mn_3_O_4_ crystal (JCPDS no.80-0382), indicating the presence of Mn_3_O_4_ in the composite material^15^.

By observing the diffraction peak of NPCS/Mn_3_O_4_@S, it can be found that the characteristic peak of the sample corresponds well with the original crystallized sulfur after sulfur injection, which indicates that sulfur is mainly covered on the surface of the hollow carbon sphere. Raman spectra of NHCS and NHCS/Mn_3_O_4_ are shown in Fig. 2b, where D region represents disordered carbon and G region represents graphitized carbon. Generally, the ratio of I_D_/I_G_ is an important parameter to measure the degree of graphitization and electrical conductivity of a substance^16^. Because NHCS/Mn_3_O_4_ has a slightly lower I_D_/I_G_ ratio (0.92 vs. 0.94), it has a better electrical conductivity than NHCS. Figure 2c displays the sample's TG curve. The sulfur load of NPCS/Mn_3_O_4_@S and PCS@S are respectively 75% and 68%, which is essentially compatible with the anticipated sulfur carrying capability. The fact that NPCS/Mn_3_O_4_@S has a higher load than PCS@S does so because of the more favorable structural characteristics of NPCS/Mn_3_O_4_. This may be due to the fact that granular and rod Mn_3_O_4_ offer more sulfur adsorption sites.

Figure 2d shows the BET related data of the two samples. The adsorption and desorption curves of NHCS and NHCS/Mn_3_O_4_ belong to Class IV adsorption isotherms, indicating that both of them have strong adsorption capacity^17^, but NHCS/Mn_3_O_4_ has a stronger adsorption capacity than PCS. It can be seen that NHCS/Mn_3_O_4_ and NHCS have similar pore size distribution, and micro mesoporous pores are distributed at 1–6 nm, indicating that the introduction of Mn_3_O_4_ does not cause large-scale damage to the pore structure. Before and after loading Mn_3_O_4_, the specific surface area is 286 m^2^ g^−1^, 304 m^2^ g^−1^, respectively. And the pore volume is 0.65 cm^3^ g^−1^ and 1.1 cm^3^ g^−1^, respectively, which may be due to the damage of the sphere during the process of Mn_3_O_4_ compound.

**Figure 3:**
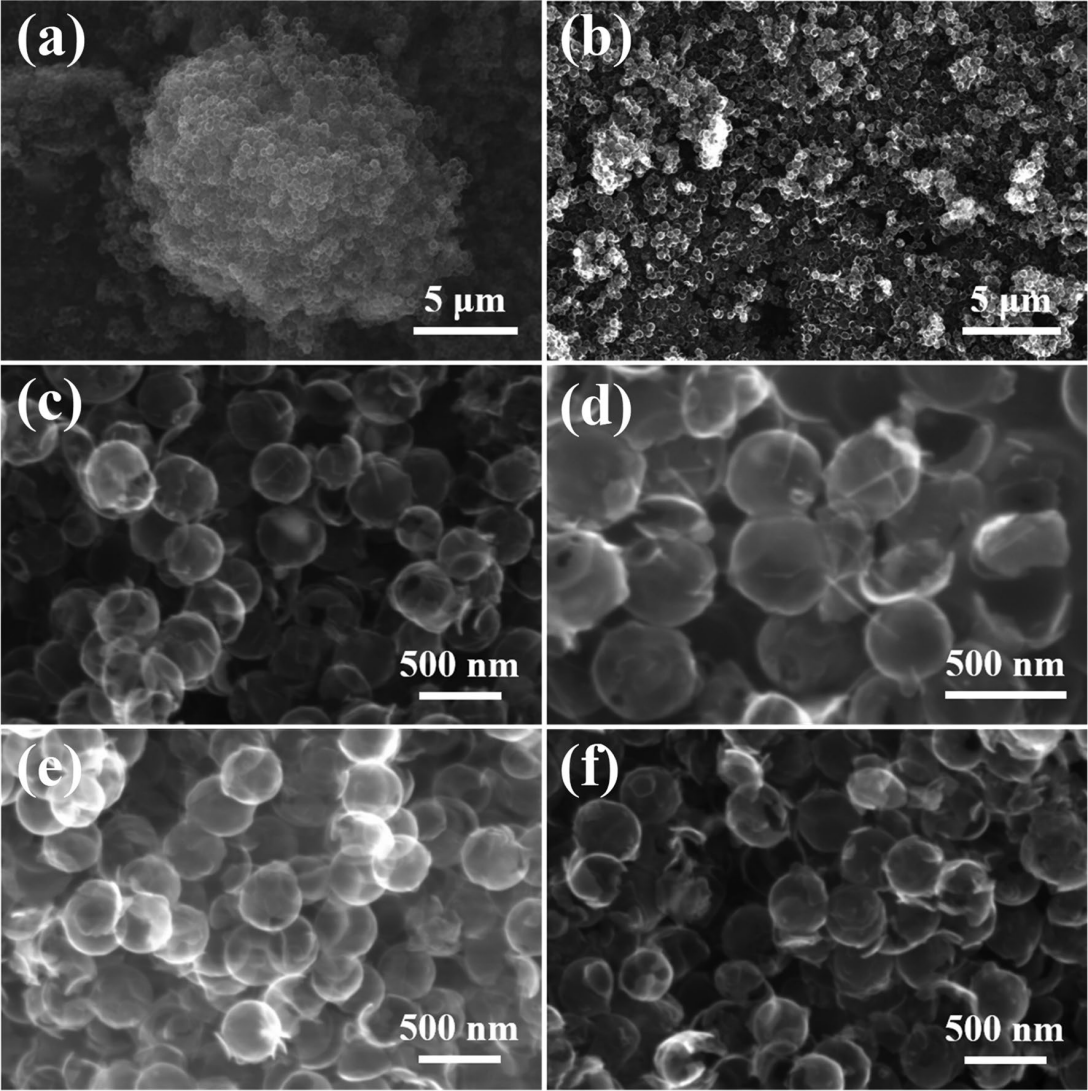
SEM figures of (**a**) NHCS, (**b**–**d**) NHCS/Mn_3_O_4_, (**e**) NHCS/S, (**f**) NPCS/Mn_3_O_4_/S.

The SEM images of the prepared sample is shown in Fig. 3. As can be seen from Fig. 3a, NHCS are uniformly distributed in the form of hollow carbon balls with a diameter of about 300 nm. Figure 3b–d shows that after the introduction of Mn_3_O_4_, there is an obvious linear distribution on the surface of the carbon sphere. Combined with the XRD data, it can be inferred that the current nano-substance is Mn_3_O_4_. In addition, the spheres of NHCS/Mn_3_O_4_ appear to be partially destroyed, which may be due to the thin carbon shell of NHCS and low mechanical strength, which is caused by ultrasound or agitation in the process of dispersing NHCS. This conclusion is consistent with the BET test results. Figure 3e shows that the transparency of the hollow carbon sphere matrix after sulfur injection is reduced, indicating that sulfur is distributed in the carbon-based material, which reduces the electrical conductivity of the composite. In addition, there is no obvious agglomeration of sulfur on the surface of the carbon sphere, and the dispersion is relatively uniform. In combination with the XRD and SEM results of NPCS/Mn_3_O_4_/S (Fig. 3f), it can be seen that the distribution of sulfur in the composite is relatively uniform, and the nanowires on the surface of NPCS/Mn_3_O_4_ disappear, which may be caused by the covering of sulfur on the surface of the carbon sphere.

**Figure 4:**
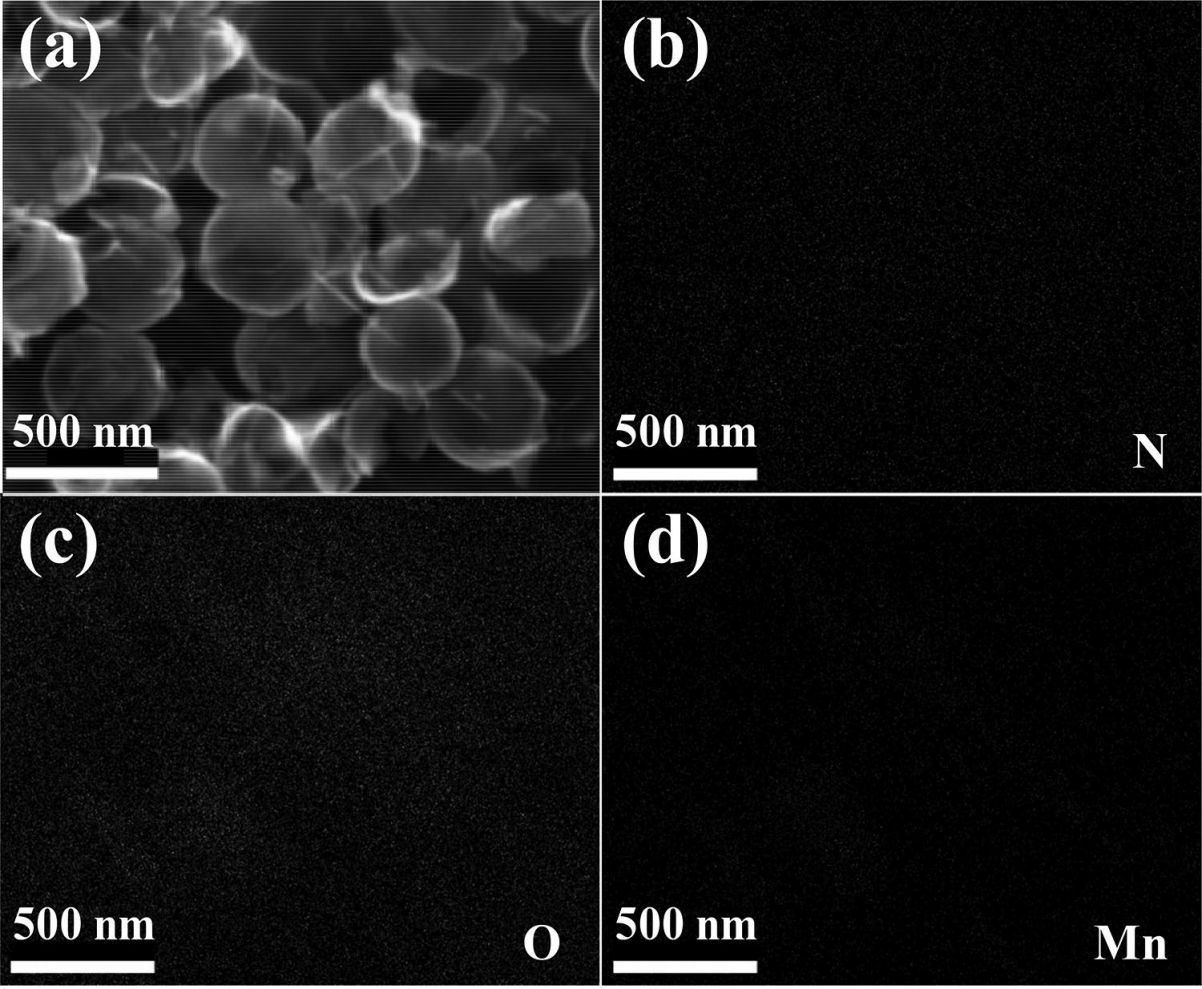
(**a**) SEM diagram of NPCS/Mn_3_O_4_; EDX diagram of (**b**) N, (**c**) O, (**d**) Mn.

According to the EDS diagram (Fig. 4b–d), it can be seen that the elements of NHCS/Mn_3_O_4_ microspheres are evenly distributed and rich in N element, because dopamine hydrochloride, as a carbon source and nitrogen source, contains a large amount of nitrogen itself, and urea is also a nitrogen source. In the distribution diagram of Mn and O elements, their brightness is stronger, which further proves that Mn_3_O_4_ grows on the hollow carbon sphere.

**Figure 5:**
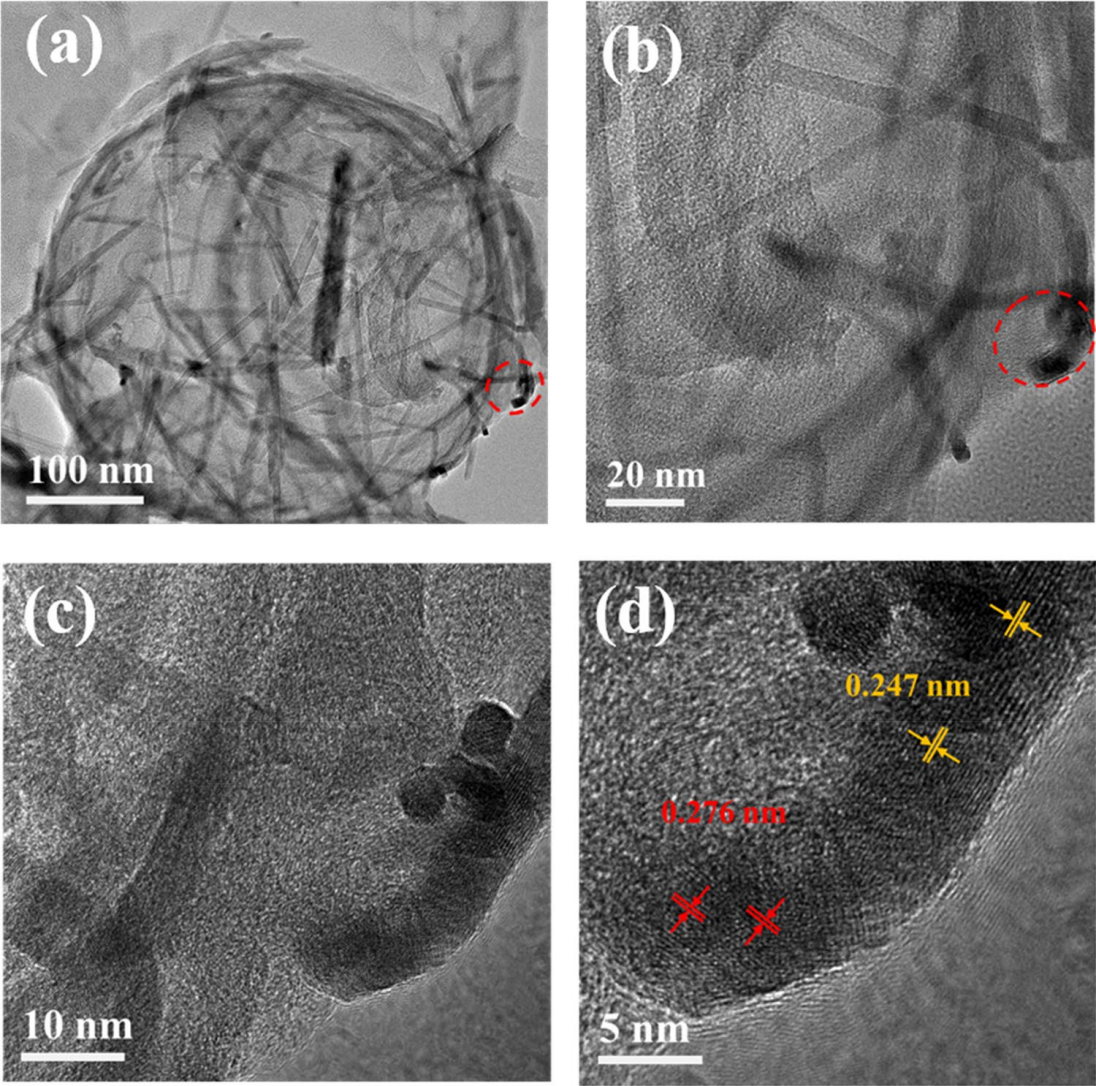
(**a**–**d**) TEM diagram of NPCS/Mn_3_O_4_.

The TEM image of NPCS/Mn_3_O_4_ is shown in Fig. 5. It can be seen that rod-shaped Mn_3_O_4_ is covered on the hollow sphere (Fig. 5a). When one of the bars of Mn_3_O_4_ is selected for high-power projection electron microscopy analysis (Fig. 5d), lattice fringes with lattice spacing of 0.276 nm and 0.247 nm can be found on it, which belong to the (1 0 3) and (2 1 1) planes of Mn_3_O_4_, respectively. The results show that Mn_3_O_4_ has been negatively bonded to the hollow carbon sphere.

**Figure 6:**
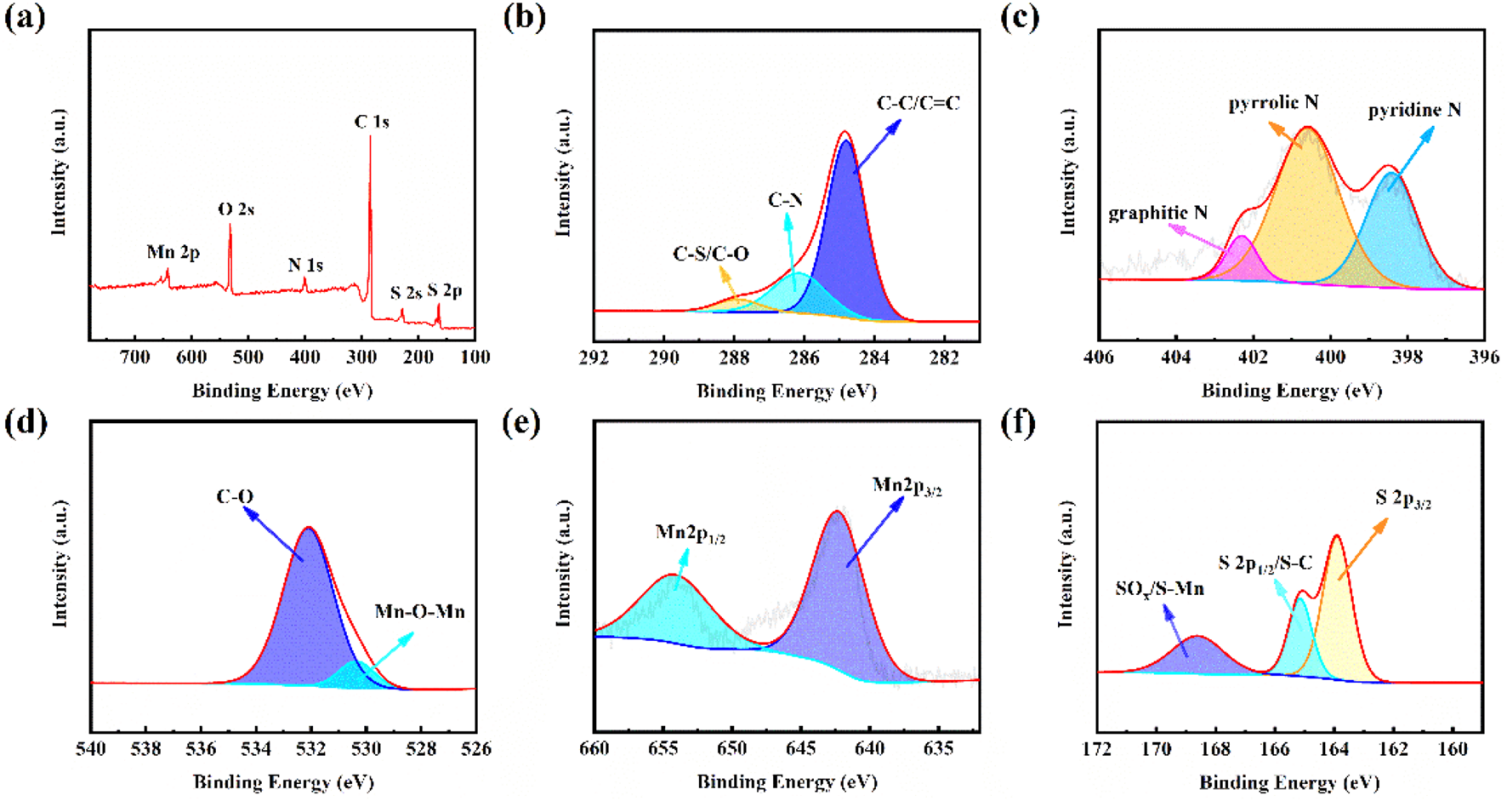
(**a**–**f**) XPS spectra of (**a**) NPCS/Mn_3_O_4_/S; (**b**) C 1s; (**c**) N 1s; (**d**) O 1s; (**e**) Mn 2p; (**f**) S 2p.

In order to further understand the chemical bonding in NPCS/Mn_3_O_4_/S, xps analysis of the composite material was performed, and the results were shown in Fig. 6. Figure 6b shows the xps fine spectrum of C 1s. Three diffraction peaks can be seen, which are C–C/C=C (284.80 eV), C–N (286.15 eV), and C–S/C–O (287.90 eV)^18^. Four peaks exists on the fine spectrum of N 1s (Fig. 6c), corresponding to Pyridinic N (398.34), pyrrolic N (400.64 eV), N–O (403.22 eV), and –NO_2_ (407.36 eV) peaks^19,20^. Peaks belonging to Mn–O–Mn (530.36 eV) can be found on the O1s fine spectrum (Fig. 6d). The band gap between Mn2p_1/2_ (654.15 eV) and Mn2p_3/2_ (642.30 eV) on the Mn 2p fine spectrum (Fig. 6e) is 11.85 eV, which further proves the existence of Mn_3_O_4_]. The peak at 642.42 eV is a satellite peak of Mn. Obvious SO_x_/S-Mn diffraction peaks can be seen on the S 2p fine spectrum in Fig. 6f, indicating the existence of Mn bonding with S, which has a positive effect on the anchored polysulfides in the cycle.

### Electrochemical properties

**Figure 7:**
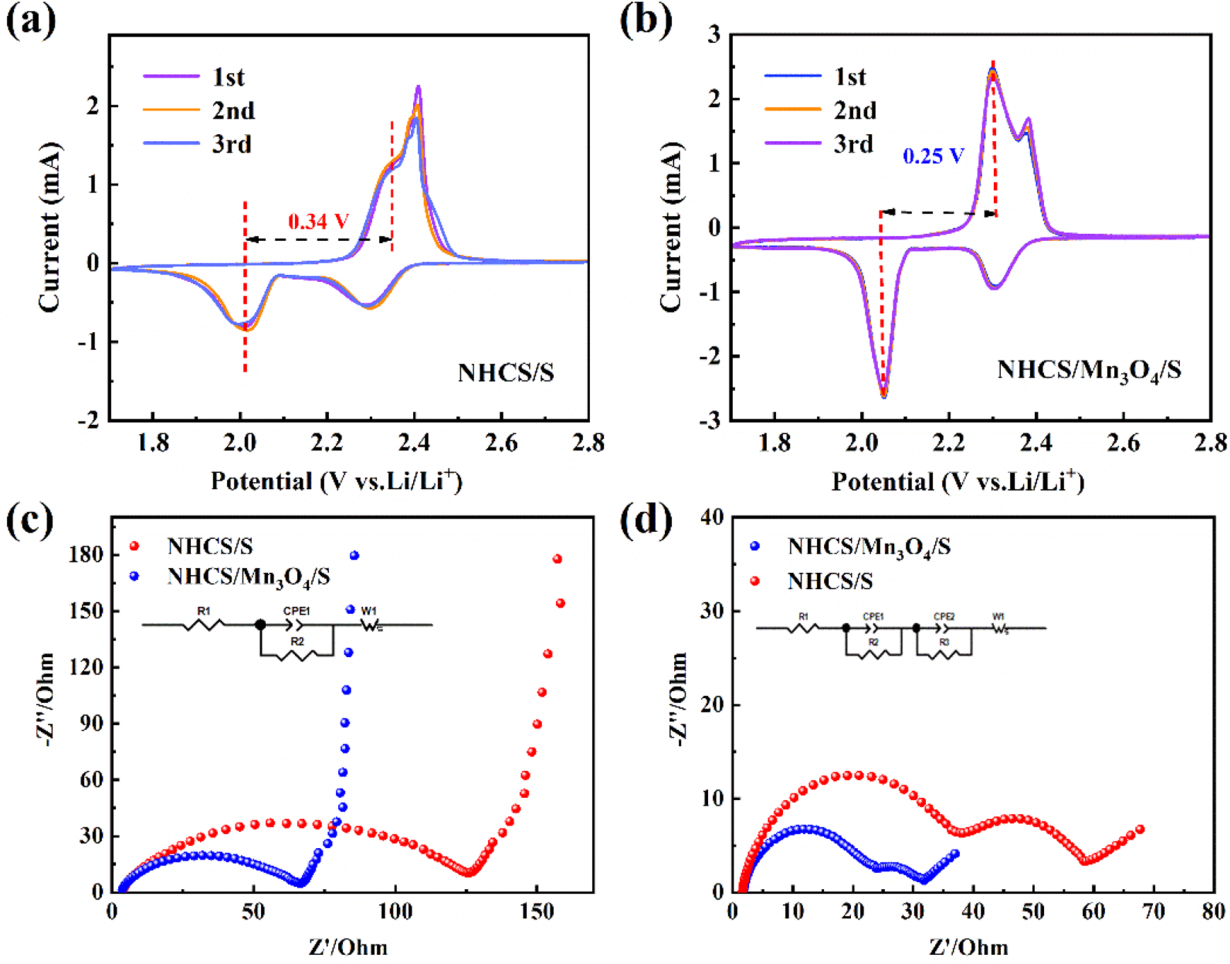
(**a**, **b**) CV curves of NHCS/S and NHCS/Mn_3_O_4_/S cathodes; (**c**, **d**) EIS spectra of NHCS/S and NHCS/Mn_3_O_4_/S batteries before 0.1 C cycle and after 100 cycles.

In order to further analyze the electrochemical properties of the sample, we assembled NHCS/S and NHCS/Mn_3_O_4_/S into a 2025 button battery for related tests. First, CV and impedance tests were carried out on the electrochemical workstation. The current density of the CV test was 0.1 mV s^−1^. The curves of NHCS/S and NHCS/Mn_3_O_4_/S cathodic cycles for the first three weeks are shown in Fig. 7a, b. It can be seen that they all have two distinct reduction peaks, corresponding to the decomposition of the S_8_ chain into short-chain lithium polysulfide and Li_2_S_2_ or Li_2_S by reaction. Correspondingly, the oxidation peak corresponds to the process of generating S_8_^21^. It is worth noting that NHCS/S only has a wide oxidation peak located near 2.35 V, while NHCS/Mn_3_O_4_/S cathode has two oxidation peaks located near 2.3 V and 2.4 V respectively, which indicates that NHCS/Mn_3_O_4_/S has obvious chemical catalysis. The partial process of polysulfide conversion to S_8_ chain is accelerated^22^.

In addition, the redox potential difference of the NHCS/Mn_3_O_4_/S electrode is significant smaller than that of the NHCS/S electrode, only 0.25 V, indicating that the NHCS/Mn_3_O_4_/S electrode has a smaller polarization, which can catalyze the adsorption and conversion of polysulfides and provide a stronger power for the redox reaction^23^. Both NHCS/S and NHCS/Mn_3_O_4_/S cathodes have good CV curve repeatability in the first three cycles, indicating that they have low electrochemical polarization and good redox reversibility^24^.

The NHCS/Mn_3_O_4_/S cathode has better adhesion, which indicates that it has better cyclic stability and stronger anchoring ability to polysulfide. On the one hand, as a polar transition metal oxide, Mn_3_O_4_ itself has a strong chemical effect on LiPSs, which can closely bind LiPSs in the complex charge and discharge process to reduce the loss of active substances caused by the shuttle effect. At the same time, Mn_3_O_4_ exists in the carbon matrix in the form of particles or rods. It can not only provide more active deposition sites for the reaction products (Li_2_S_2_ or Li_2_S), provide physicochemical power for the adsorption and transformation of lithium ions, but also shorten the transmission distance of ion electrons, accelerate the reaction process, and thus show a smaller polarization^25,26^.

AC impedance test was performed on the battery before cycling and after 50 cycles at 0.1 C current density, and the results were shown in Fig. 7c, d. The Nyquist curve is the most common way to represent the impedance of a button cell and is usually composed of a semicircle in the high frequency region and a slash in the low frequency region. The high-frequency semicircle is related to charge transfer resistance (R_ct_) and the diameter of the semicircle is the resistance value. The slash line in the low frequency region is related to the diffusion resistance (R_e_), and generally the higher the slope, the lower the viscosity of the electrolyte and the lower the resistance of ion diffusion. The intersection of the starting point and the real axis is related to the electrolyte resistance^27^.

After using ZView software to simulate the equivalent circuit to fit the corresponding impedance values, the results obtained are shown in Table 1. The Re and R_ct_ of NHCS/S and NHCS/Mn_3_O_4_/S cathodes before cycling are 2.7 Ω and 2.6 Ω, respectively, and 113.6 Ω and 61.1 Ω, respectively, indicating that NHCS/Mn_3_O_4_/S cathodes have smaller interface impedance and charge transfer resistance values. The increased semi-circle in the middle and high frequency region of the impedance spectrum after the cycle is due to the impedance (R_s_) generated by the deposition of reaction products Li_2_S and Li_2_S_2_ on the electrode surface to form SEI film (solid electrolyte film) during the cycle. It can be found that after 50 cycles, the RCTS of NHCS/S and NHCS/Mn_3_O_4_/S cathodes decrease to 30.8 Ω and 16.2 Ω, Rs are 26.2 Ω and 11.5 Ω, and R_e_ are 1.6 Ω and 1.5 Ω, respectively. The decrease of Rct resistance indicates that the conductive layer effectively inhibits the shuttle of LiPSs and accelerates the interfacial charge transfer^28,29^. The resistance values of NHCS/Mn_3_O_4_/S cathode before and after cycling are smaller than NHCS/S, indicating that it has higher conductivity, which is consistent with Raman results.

**Table 1:** Impedance parameters in equivalent analog circuit (Ω).

	Resistance	NHCS/S	NHCS/Mn_3_O_4_/S
Before 1 cycle	R_e_	2.7	2.6
	R_ct_	113.6	61.1
After 100 cycles	R_e_	1.6	1.5
	R_ct_	30.8	16.2
	R_s_	26.2	11.5

**Figure 8:**
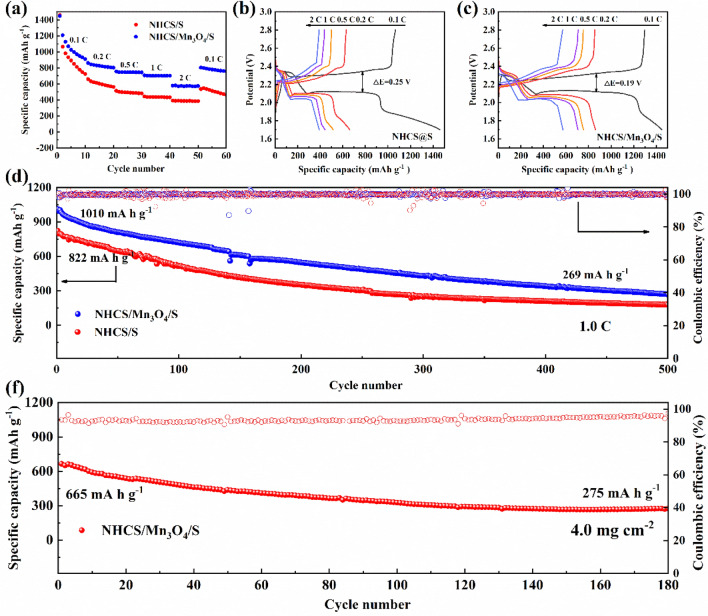
(**a**, **b**) Rate properties of NHCS/S and NHCS/Mn_3_O_4_/S cathodes; (**b**, **c**) the first charge and discharge curves of NHCS/S and NHCS/Mn_3_O_4_/S cathodes at 0.1 C, 0.2 C, 0.5 C, 1.0 C and 2.0 C; (**d**) cycle performance and coulomb efficiency at 1.0 C; (**f**) cycle performance and coulomb efficiency at the sulfur load reaches 4.0 mg cm^−2^.

Figure 8a shows the power performance of NHCS/S and NHCS/Mn_3_O_4_/S cathodes, and Fig. 8b, c shows the corresponding charge and discharge curves under different current densities. The magnification performance shows that the NHCS/Mn_3_O_4_/S cathode has an initial specific discharge capacity of 1447.1 mAh g^−1^ at 0.1 C. Under the current density of 0.1 C, 0.2 C, 0.5 C, 1.0 C and 2.0 C, the average specific discharge capacity during 10 cycles was 1064.5, 826.3, 747.8, 702.1 and 574.2 mAh g^−1^, respectively. Always higher than the NHCS/S cathode 924.6, 602.7, 493.2, 436.9, 388.7 mAh g^−1^, in addition, when the current density returned to 0.1 C, NHCS/Mn_3_O_4_/S cathode maintained a high reversible capacity of 805 mAh g^−1^, Significantly higher than the NHCS/S cathode of 537.5 mAh g^−1^. The above data fully show that the NHCS/Mn_3_O_4_/S cathode has better magnification performance, higher utilization of active substances, and faster current response ability. By observing the results of Fig. 8b, c, it can be found that the cathode presents an obvious charging and discharging platform at different current densities. It is worth noting that the redox potential difference of the NHCS/Mn_3_O_4_/S cathode is smaller than that of the NHCS/Mn_3_O_4_/S cathode, which is only 0.19 V, indicating that the NHCS/Mn_3_O_4_/S cathode has a smaller polarization phenomenon. Stronger R redox reaction kinetics.

In order to analyze the long cycle performance of different composites, the NHCS/S and NHCS/Mn_3_O_4_/S cathodes were tested for 500 cycles at a high current density of 1.0 C, and the results were shown in Fig. 8d. The activated NHCS/Mn_3_O_4_/S cathode showed a high initial discharge specific capacity of 1010 mAh g^−1^ at a current density of 1.0 C, and still maintained a reversible capacity of 269 mAh g^−1^ after 500 charge and discharge cycles. The initial specific discharge capacity of NHCS/S cathode is only 822 mAh g^−1^, and the capacity during the long cycle is always lower than that of NHCS/Mn_3_O_4_/S. Obviously, NHCS/Mn_3_O_4_/S cathodes show better long cycle performance. However, the initial discharge specific capacity of the Mn3O4/S cathode at 1.0 C is only 583 mAh g^−1^, and the charge and discharge are unstable, which is caused by the fixation of the active substances by less NHCS. The excellent rate performance and long cycle stability are in line with expectations and are consistent with CV and EIS results, thanks to the successful structural design of the NHCS/Mn_3_O_4_/S cathode, which has a synergistic effect of physical adsorption and chemisorption and catalysis. The nitrogen-containing hollow carbon sphere not only has a large cavity structure, but also can absorb polysulfide polarities. Therefore, both composites exhibit high initial discharge specific capacity. In addition, Mn_3_O_4_ can effectively anchor LiPSs and prevent soluble LiPSs from being dissolved in the electrolyte, thus improving the utilization rate of sulfur. The chemical catalysis of Mn_3_O_4_ can also provide a stronger driving force for the transformation of polysulfides and accelerate the reaction process^30,31^. Therefore, NHCS/Mn_3_O_4_/S cathodes exhibit faster rate response and long cycle stability. As shown in Fig. 8f, when the sulfur load reaches 4.0 mg cm^−2^, NHCS/Mn_3_O_4_/S has an initial specific capacity of 665 mAh g^−1^, and still has a reversible specific capacity of 275 mAh g^−1^ after 180 cycles. This shows that the composite material can still carry out a certain number of cycles under high sulfur load.

**Figure 9:**
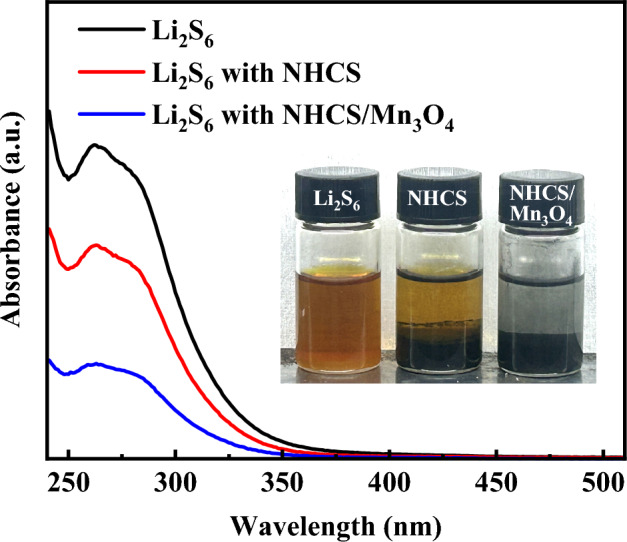
Ultraviolet–visible absorption spectra of Li_2_S_6_ solution and NHCS and NHCS/Mn_3_O_4_ solution after standing for one hour and their photos.

Further, in order to characterize the adsorption capacity of the material for polysulfide, we conducted adsorption experiments, and the relevant results are shown in Fig. 9. The Li_2_S_6_ solution is prepared in the glove box, and its color is dark orange. A part of it is taken in the glass bottle as the control, and then the NHCS and NHCS/Mn_3_O_4_ powder of the same quality are added to the Li_2_S_6_ solution of the same volume to adsorb the polysulfide. After standing for one hour, the color change is observed and photographed. It can be found that the solution containing sample NHCS/Mn_3_O_4_ is nearly colorless, and the solution containing NPCS is light yellow, indicating that NHCS/Mn_3_O_4_ has a stronger adsorption capacity and absorbs more Li_2_S_6_. Uv–vis absorption spectra of the above solutions were tested, and the detection results showed that the absorption peak corresponding to NPCS/Mn_3_O_4_ material was the lowest, which was consistent with the experimental results, which further explained the contribution of Mn_3_O_4_ in the composite material to the adsorption of polysulfide.

## Conclusion

In this work, nitrogen-containing hollow carbon spheres with an average diameter of about 300 nm were prepared by template method, and polar Mn_3_O_4_ was introduced into the composite material by in-situ growth as a multifunctional sulfur host. Mn_3_O_4_ is distributed on the surface of hollow carbon spheres in the form of nanowires. After the introduction of Mn_3_O_4_, a small part of the carbon sphere structure is destroyed, possibly due to insufficient mechanical strength during the process of stirring or ultrasonic dispersion of the carbon sphere. Electrochemical test results show that the host not only has high electrical conductivity, but also can give full play to the advantages of physical and chemical coordination, and achieve a good electrochemical catalytic effect, which shows a high initial discharge specific capacity, fast rate response ability, long life and cycle stability in LSBs. The activated NHCS/Mn_3_O_4_/S cathode showed a high initial discharge specific capacity of 1010.3 mAh g^−1^ at a current density of 1.0 C, and still maintained a reversible capacity of 269.2 mAh g^−1^ after 500 cycles. The initial specific discharge capacity of NHCS/S cathode is only 822.6 mAh g^−1^.

## Data Availability

The datasets used and analyzed during the current study are available from the corresponding author on reasonable request.
